# Feature Reduction for Molecular Similarity Searching Based on Autoencoder Deep Learning

**DOI:** 10.3390/biom12040508

**Published:** 2022-03-27

**Authors:** Maged Nasser, Naomie Salim, Faisal Saeed, Shadi Basurra, Idris Rabiu, Hentabli Hamza, Muaadh A. Alsoufi

**Affiliations:** 1 School of Computing, Universiti Teknologi Malaysia, Johor Bahru 81310, Malaysia; idrisrabiu43@gmail.com (I.R.); hentabli_hamza@yahoo.fr (H.H.); muaadh.soufi2021@gmail.com (M.A.A.); 2 DAAI Research Group, Department of Computing and Data Science, School of Computing and Digital Technology, Birmingham City University, Birmingham B4 7XG, UK; shadi.basurra@bcu.ac.uk; 3 Laboratory of Advanced Electronic Systems (LSEA), University of Medea, Medea 26000, Algeria

**Keywords:** molecular similarity, drug design, autoencoder, irrelevant and redundant features

## Abstract

The concept of molecular similarity has been commonly used in rational drug design, where structurally similar molecules are examined in molecular databases to retrieve functionally similar molecules. The most used conventional similarity methods used two-dimensional (2D) fingerprints to evaluate the similarity of molecules towards a target query. However, these descriptors include redundant and irrelevant features that might impact the performance of similarity searching methods. Thus, this study proposed a new approach for identifying the important features of molecules in chemical datasets based on the representation of the molecular features using Autoencoder (AE), with the aim of removing irrelevant and redundant features. The proposed approach experimented using the MDL Data Drug Report standard dataset (MDDR). Based on experimental findings, the proposed approach performed better than several existing benchmark similarity methods such as Tanimoto Similarity Method (TAN), Adapted Similarity Measure of Text Processing (ASMTP), and Quantum-Based Similarity Method (SQB). The results demonstrated that the performance achieved by the proposed approach has proven to be superior, particularly with the use of structurally heterogeneous datasets, where it yielded improved results compared to other previously used methods with the similar goal of improving molecular similarity searching.

## 1. Introduction

Virtual Screening (VS) is one of the most extensively utilized computational methods for searching for small molecule libraries in drug discovery. The vs. is often used to discover structures most likely used as binding for a drug target [1]. In virtual screening, there are two approaches: ligand-based virtual screening (LBVS) and structure-based virtual screening (SBVS). Similarity searching is one of the LBVS approaches that is used to search and scan chemical databases for molecules that are most similar to a user-defined reference structure using a quantitative measure of intermolecular structural similarity. LBVS methods search for molecules structurally similar to the ligand and need a known active input. The second approach, SBVS, searches for compounds that match the target binding site and needs the target protein’s structure [2]. The basic underlying assumption in similarity searches is that structurally similar compounds would have similar physicochemical and biological properties [3]. 

The 2D similarity methods are the most commonly used for large numbers of molecules. The concept behind the molecular similarity measure is that molecules with similar structures have a higher degree of similarity than molecules with diverse structures. Therefore, the goal of similarity searching is to retrieve molecules that exhibit a structural similarity with the user’s reference structure. Scaffold Hopping is a term used in chemoinformatics to refer to the process of identifying structurally diverse molecules that exhibit biological activity. This process may be used to learn more about compounds that have been discovered as active compounds by modifying the molecule’s core structure. Hence, newer methods for identifying biologically active compounds must be developed [4,5].

As discussed above, measuring the similarity of two molecules is highly important and is routinely performed in chemoinformatics. Various coefficients measure the degree of similarity/dissimilarity between two molecules. The basic concept of determining similarity/dissimilarity based on numerical measures has been widely implemented in various areas. However, the lack of communication between these disciplines presents an opportunity to reinvent similar coefficients under different names, leading to duplication [6,7]. 

The ability of a coefficient to accurately predict the property/activity value of a compound is used to determine its effectiveness, which can be determined using the values of the most similar compounds in the same dataset. Many subsequent studies compared the effectiveness of various similarity coefficients and concluded that the Tanimoto coefficient surpassed the others [8]. Thus, the Tanimoto coefficient has been acknowledged as the standard similarity measure of chemical compounds in chemo-informatics [9].

The majority of currently used similarity-based virtual screening methods deal with massive amounts of data that contain redundant and irrelevant features. The present molecule’s fingerprint consists of several features. Furthermore, due to the irregularities in their relevance levels, removing some of the features may improve the recall of the similarity measure [10]. Data with irrelevant and redundant features might mislead the virtual screening findings and make them harder to interpret [11,12]. Numerous modern fingerprints are complex, consisting of many features as well as many bit locations, with typically over 1000 features.

Deep learning (DL) techniques based on deep artificial neural networks have greatly advanced state-of-the-art in computer vision [13,14], speech recognition [15,16], natural language processing [17,18], and molecular bioactivity prediction [19]. Deep learning has made considerable gains, bringing it closer to one of its main goals: Artificial Intelligence. One advantage of DL is that it is beneficial for feature learning, which can be done automatically using a general-purpose technique. This procedure is frequently used by implementing a multi-layer stack of simple neural networks with non-linear input-output mappings, including deep neural networks (DNNs) [20], convolutional neural networks (CNNs) [21,22], recurrent or recursive neural networks (RNNs) as well as deep networks with more than one hidden layer and more neurons in each layer. Besides, DL architectures have proven capable of handling large volumes of data with minimum manual intervention [23].

Over the last several years, DL technology has advanced dramatically. This approach outperformed other ML algorithms in terms of empirical findings, most likely because, similar to the brain model, this method replicates brain activity by stacking multiple neural network layers [24,25,26]. According to Wang and Raj [27], who use the feature extraction approach, DL methods outperform conventional machine learning methods. However, there is no theoretical underpinning for DL technology at this time. The DL approaches are used to learn feature hierarchies by combining features from higher hierarchical levels with low-level features. The availability of feature learning at different abstraction levels enables the system to learn sophisticated functions that map input and output from data without requiring human-developed features [27]. Handcrafted features are extracted and fed into SVM and other classification algorithms in the conventional setup for image recognition systems. On the other hand, deep learning outperforms conventional methods since it optimizes all extracted features.

The most noticeable distinction between machine learning and deep learning technologies is how their effectiveness fluctuates as the data increases. DL techniques perform inefficiently on smaller datasets as they require a large amount of data to comprehend adequately [28].

Autoencoders (AE) are a sophisticated deep learning technique used in situations involving complicated data such as images and videos. AE is good at handling low dimensional feature representation from the inputs based on unsupervised learning [29,30,31]. The AE has the benefit of providing a functional relationship between the high-dimensions and low-dimensions representations, as well as vice versa. The AE establishes efficient functional links between the high-dimensions and low-dimensions representations and is compelled to offer a meaningful point arrangement in the low-dimensions representation by employing a non-linear distance metric-based cost function [32]. In chemoinformatics, one of the major drawbacks in chemical fingerprints in virtual screening is that the fingerprint descriptors often consist of irrelevant and redundant features, and removing some of these features can improve the recall of the similarity measure performance [10]. In this paper, a new similarity-based virtual screening approach has been developed based on a new molecular representation that uses Autoencoder to remove irrelevant and redundant features to provide low dimensions. This new representation with low dimensions is regarded as a new descriptor and utilized to enhance the recall of the similarity searching measures. Based on the experiments conducted, the results demonstrated that the new proposed representation based on Autoencoder is effective and superior to the proposed benchmarks methods using full descriptors features. In general, this paper presents the following significant contributions:

- Proposing a novel ligand-based virtual screening dimensionality reduction method based on Autoencoder deep learning offers low-dimensional representations of molecular features while removing irrelevant and redundant features that affect similarity searching.
- Enhancing the effectiveness of the similarity searching by applying the proposed low dimensional representation of molecules.
- The proposed method has demonstrated superior results in terms of overall performances than the benchmark methods, e.g., TAN, ASMTP, and SQB.

## 2. Related Work

Many similarities between text information retrieval and chemoinformatics have suggested that techniques developed in text documents information retrieval can perhaps be used to enhance similarity searching of molecules [33]. Hence, many molecular similarity approaches employed in ligand-based virtual screening were originally based on the text retrieval domain. Bayesian Inference Networks are one of the techniques that have been extensively used for text in a variety of domains, as well as substantially used in virtual screening as a substitute for conventional similarity searching strategies, surpassing conventional similarity approaches [34,35,36,37]. Several similarity measures have been recently developed for virtual screening that outperformed the Tanimoto coefficient, such as quantum-based similarity measure (SQB) [38] and adapting text similarity measures (ASMTP) [39] which has been derived from a similarity measure of text processing and ideal for virtual screening.

The fragment bases and bit-strings similarity method has gained attention from researchers in chemoinformatics and especially in virtual screening [40,41]. The weight of each fragment in chemical structure compounds has been examined by adding more weight to highly significant fragments [42]. In Ligand-Based Virtual Screening, several weighting functions have been presented for a new fragment weighting approach for Bayesian Inference Network [40]. The fragment reweighting approach was developed by integrating reweighting variables with relevance feedback to enhance the Bayesian Inference Network’s retrieval recall performance [43]. 

Several reweighting methods that have been used, such as features reweighting, features selection, mini-fingerprint, and fuzzy correlation coefficient, have been used to improve the performance of the similarity methods [44,45,46]. However, performance over a highly diverse dataset is still low and requires more enhancement [41]. 

Data fusion approaches have significantly improved the overall performance of conventional similarity algorithms [47,48]. They combine multiple data sources into a single source, with the output of the combined source expected to be more informative than the input sources individually [49,50]. Most chemical representations, query molecules, docking scores, and similarity coefficients were integrated using linear combination techniques [51]. Many fusion studies, whether in text or chemical compound retrieval, have established that using multiple sources rather than a single source yields a greater outcome. To enhance retrieval performance via data fusion, two requirements must be met: the accuracy of each source and the independence of sources [52]. 

Samanta et al. [53] introduced a novel approach for molecular similarity, in the form of a variational autoencoder (VAE) to instate a new method that uses only the canonical SMILES encoding of the molecules themselves, leading to its representation as a 100-element vector and using Simple Euclidean distances to obtain a metric of similarity calculated for any new molecule, including the entire set of molecules used in the development of the latent space. First, The VAE has been trained to use SMILES molecule representation. This training returned more than 95% valid SMILES in the test (holdout) set, so those that were invalid could simply be filtered out without significant loss of performance. Following training, each molecule (SMILES) was associated with a normalized vector of 100 dimensions, and the Euclidean distance between them was calculated. The VAE was trained on over six million druglike molecules and natural products (including over one million in the final holdout set). The VAE vector distances provided a rapid and novel metric for molecular similarity that is easily and rapidly calculated. The new metrics determine the similarity to clozapine of other drugs. 

Recently, Nasser et al. [54] developed a new feature selection model based on the deep belief networks method for ligand-based virtual screening. The reconstructed features weight and features error were calculated, and the features were filtered according to the value of the features error. Important features with lower error values are selected based on threshold and utilized to enhance the recall of similarity searching measures [54,55]. Another recent new research focuses on determining whether some descriptors and molecular presentation are better as individual or complementary to other descriptors. The results show that the combined use of descriptors demonstrated to be better [56]. Several molecular representations, in particular, are included by merging and integrating features from multi-descriptors, which improves the effectiveness of similarity searching [56]. The proposed research was based on a new feature selection model based on deep belief networks applied to five descriptors, with the significant features from each descriptor being selected and integrated to generate a new descriptor. This descriptor is then used to improve the final performance of molecule similarity searching [56,57].

## 3. Materials and Methods

### 3.1. Dimensionality Reduction Based Autoencoder

Autoencoder (AE) is a method that encodes some input into a low-dimensionality representation known as code and then reconstructs this compact representation to match the original input as closely as possible using a decoding module [58]. AE is obliged to learn an encoding transformation that contains the most important information about the structural data for the decoding component to function effectively in the reconstruction task. The AE is divided into two parts: an encoder and a decoder. The encoder takes the original input and makes a limited representation of it, referred to as the representation code layer or the latent space layer, while the decoder is in charge of reconstructing the original input from the code layer. Encoders and decoders are frequently linear transformations that may be done unsupervised using a dense layer of a neural network [59].

**Figure 1:**
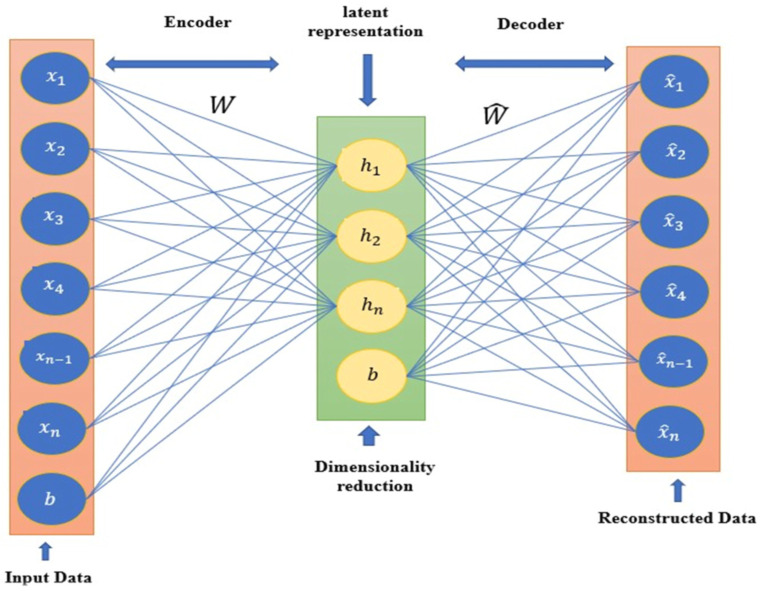
The Standard structure of Autoencoder.

The AE transforms a molecule into a continuous space, which the decoder then utilizes to rebuild the molecule based on its continuous representation. Therefore, by employing this fundamental concept, the model is not required to acquire a generic mathematical representation of the molecules. Due to the large number of parameters in Neural Networks and the relatively lesser amount of training data, the AE will almost certainly learn an explicit mapping of the training set, and the decoder will be unable to decode random points in the continuous space [60]. Figure 1 depicts a basic autoencoder architecture. The encoder takes x input to a hidden representation h and a decoder which reconstructs the input $\hat{x}$ back from the h. 

An encoder is a deterministic mapping function f(x) that converts a d-dimensional input vector x into an r-dimensional hidden representation h called an encoder [61]. It commonly takes the form of an affine mapping preceded by a nonlinearity, as seen in the following:

(1) h=f(x)=Φ(wx+b)=sigmoid (wx+b)=11+e−(wx+b)

where w denotes the affine mapping weight matrix, b denotes the bias vector and Φ is the activation function, typically a non-linear squashing function known as the sigmoid function. 

A decoder is a mapping function g(h) that converts the latent representation h derived based on Equation (3) into a reconstructed vector z in the input space. A decoder can alternatively take the form of an affine mapping with a squashing nonlinearity [61], which can be stated as the following:

(2) z=g(h)=Φ(w^h+b^)=sigmoid (w^h+b^)=11+e−(w^h+b^)

where $\hat{w}$ and $\hat{b}$ are the affine mapping weight matrix and the bias vector, respectively, and Φ(h) is the activation function known as the sigmoid function. 

**Figure 2:**
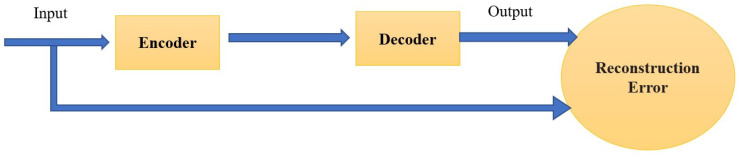
The visualization description of Autoencoder.

Generally, learning in autoencoders includes the optimization of the weights for the minimization of the reconstruction error. Hence, the objective function can be expressed as the following:

(3) $$ℒ={\left\|x-\hat{x}\right\|}^{2}$$

which is the mean squared error (MSE) between the input data and the reconstructed data and the tied weights is commonly used, i.e., $w={\hat{w}}^{T}$ [62]. Figure 2 shows the structure and the visualization description of an autoencoder.

### 3.2. Ability of Autoencoder for Molecular Dimensionality Reduction

A certain level of dimensionality is required to retrieve useful information such as important states and major conformational shifts. The ability of dimensionality reduction methods varied to efficiently project huge amounts of data to useful low-dimensional (low-d) representations varied, as did the manner the low-d and high-dimensional (high-d) representations are connected [32]. The autoencoder method has the benefit of establishing a functional link between the high-d and low-d representations and vice versa. This allows us to not only effectively project data points to a low-d representation but also to generate high-d representations for each point on the low-d map. The Autoencoder creates efficient functional links between the high-d and low-d representations. It is compelled to offer a meaningful point arrangement in the low-d representation by employing a non-linear distance metric-based cost function.

In this paper, deep Autoencoder is proposed to exploit the powerful ability to learn a feature representation of molecules from low-level encodings of a large corpus of chemical structures. It employs the concepts of neural machine translation to translate between two semantically similar but syntactically diverse representations of chemical structures, condensing the relevant information shared by both representations into a low-dimensional representation vector. After training the model, this molecule’s representation can be retrieved and utilized as a new descriptor for similarity searching. The Tanimoto similarity measure was used to search for molecular similarity using the new low-dimension descriptor. The findings were compared to existing similarity approaches that utilized the original full-dimension descriptor.

**Figure 3:**
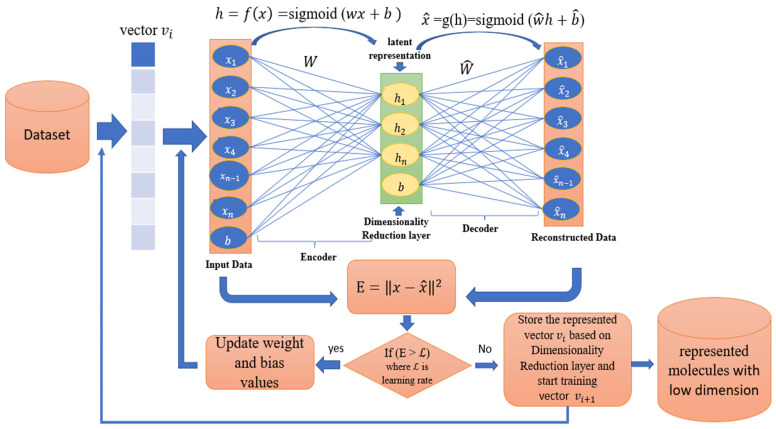
Autoencoder framework for molecular dimensionality reduction.

Figure 3 depicts the general framework of the Autoencoder proposed method for molecular dimensionality reduction, which begins by training the Autoencoder to calculate the weight of the reconstructed feature for the molecule, then calculates the mean squared error by subtracting the input molecule features values from the weight of the reconstructed features. If the mean squared error value for the trained molecule is greater than the proposed autoencoder learning rate, the autoencoder matrix weight and bias weight should be updated, and the Autoencoder trains again till the mean squared error becomes less than the proposed learning rate. The new representation of molecules based on the code layer is then saved. A similar process is conducted on all proposed datasets molecules. The output of this training is a new molecules representation with low dimensions based on the size of the latent space code layer (autoencoder dimension reduction layer).

Algorithm 1 shows the pseudo-code used to represent the proposed datasets molecules, which is based on an autoencoder with a variable number of encoder and decoder layers to build a new low-dimensional molecules representation dependent on the size of the latent space code layer.
**Algorithm 1:** Autoencoder Algorithm. The Pseudocode of the proposed Autoencoder algorithm for 2D molecular fingerprints.1: Mols = 2 D figerprints dataset descriptor2: M = number of database molecules.// 1025163: N = number of hidden layers.4: α = learning rate value.5: Epoch = 0;6: **For** k = 1:M   // for all dataset molecules7:  Input = Mlos(k)    // input data8:  x = mols(k); // initial the encoder input layer with molecule k.9:  **AE(x)**   // **Autoencoder function**10:    **For** i = 1 until N   //**start the encoder phase**11:        **If** epoch = 0 do // first time training12:        w_i_ = random (0,1) // initial the weigh matrix for the first time training13:        b_i_ = random (0,1) // initial the bias vector for the first time training14:      **Else**15:        $w_{i}=w_{i}+ℒ$ // update the weigh matrix based on the error value16:        $b_{i}=b_{i}+ℒ$ // update the bias vector based on the error value17:        hi=11+e−(wix+bi) //calculate the hidden layers values based on Equation (1)18:      x = h_i_   // make the hidden layer values to be an input to the next hidden layer.    **End**   // **end encoder phase**19:   Encoded date = x     //keep the last encoder layer which the new represented molecule.20:   h = x          //keep last encoder layer be an input encoder layer.21:   n = N22:   **For** j = 1 until N    //**start decoder phase**23:      ${\hat{w}}_{j}$ = w_n_^T^   //male the weight matrix of the decoder layer j be the transpose of n encoder weight matrix layer24:      ${\hat{b}}_{j}={{\hat{b}}_{n}}^{T}$  // make the value of the bias vector of the decoder layer j be the transpose of bias vector of n encoder layer.25:      zj=11+e−(w^jh+b^j)  // calculate the z_j_ reconstructed decoder layer values26:      h = z_j_; // keep the hidden decoder layer values to be an input to the next hidden layer.27:      n = n−1   **End**       // **end decoder phase**28:  output = h     // Reconstructed data29:  $ℒ=\|\mathrm{input}-{\mathrm{output}\|}^{2}$   // calculate the error value based on Equation (3)30:  **If** (ℒ>α)         // if the error value is greater than the learning rate.31:   Epoch = epoch+1     // need more training to reduce the error value32:   Go to 9       // call the AE function again for new training—fine tune.   **Else**33:   New_Rep_mols(k) = Encoded date;//

### 3.3. Autoencoder Proposed Cases for Molecular Dimensionality Reduction

In the paper, three different architectures of Autoencoder are proposed, namely AE1-DR, AE2-DR, and AE3-DR. Each Autoencoder consists of a different number of encoder and decoder layers while using a different number of layer nodes for molecular representation. The three proposed cases of AE are trained using different error rates (0.01, 0.05, 0.06) and different epochs (20, 30, 50, 70, 100), and for each we calculated the similarity of the molecules based on the new proposed molecular representations. The performance of the similarity searching methods was compared with other benchmarks methods. The experiments showed that the best results were obtained when the epoch is 100 and the error rate is 0.01. These three proposed cases are further explained in the following subsection. 

#### 3.3.1. Proposed Autoencoder Case 1 (AE1-DR)

In this case, the Autoencoder is trained using four encoder layers. The first encoder layer is known as the input layer, which consists of 1024 nodes and 1024 features of the extended connectivity fingerprints count (ECFC) for each molecule in the datasets. The remaining three hidden layers have 900, 700, and 500 nodes, respectively. The last hidden layer of the encoder is called the code layer (encoded data). The size of this vector is 500 dimensions which will be used as a new molecule’s representation. The decoder is a reconstruction of the encoder representation where the nodes for all the decoder layers in this care are 500, 700, 800, and 1024, respectively. The last decoder layer is known as the output layer, which is the reconstructed input data most like the input data. 

**Figure 4:**
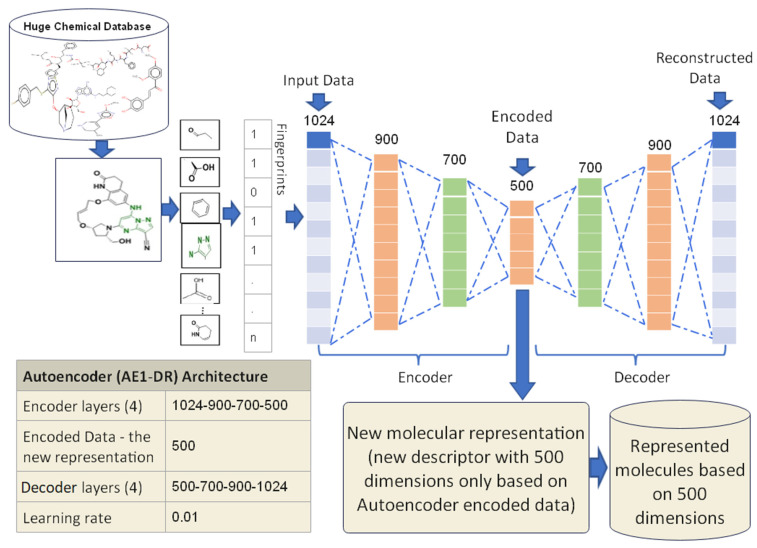
The AE1-DR proposed design.

Each molecule in the proposed datasets has been trained using AE1-DR until the error value of the training became lesser than the proposed learning rate value, which is 0.01. The new representation of molecules based on the code layer is then saved. A similar process has been conducted on all proposed datasets molecules. The proposed design of AE1-DR is shown in Figure 4. The output of this proposed case of Autoencoder is a new molecule representation with 500 dimensions only used for similarity searches between molecules. The experimental results of AE1-DR have been presented in Section 5.

#### 3.3.2. Proposed Autoencoder Case 2 (AE2-DR)

**Figure 5:**
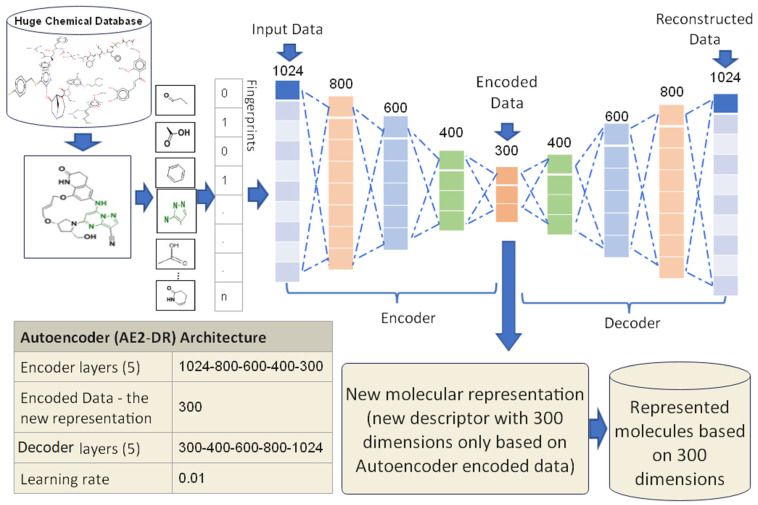
The AE2-DR proposed design.

The AE2-DR has been trained using five encoder layers in which the input data to the first layer of the encoder is the molecule vector with 1024 features. The remaining four hidden layers have 800, 600, 400, and 300 nodes, respectively. The size of the encode layer in this proposed case is 300, where it will be used as a new representation for the molecules and the size of the decoder layers in this proposed case are 300, 400, 600, 800, and 1024, respectively. The last decoder layer is the reconstructed input data which is mostly like the input data. All the molecules in the proposed datasets have been trained using the AE2-DR until they achieved a lesser error rate of the training compared to the proposed learning rate value, which is 0.01. The proposed design of AE2-DR is presented in Figure 5. The finished training output of AE2-DR is a new molecules representation with 300 dimensions which is only used for similarity search between molecules. The experimental results of AE2-DR have been presented in Section 5.

#### 3.3.3. Proposed Autoencoder Case 3 (AE3-DR)

**Figure 6:**
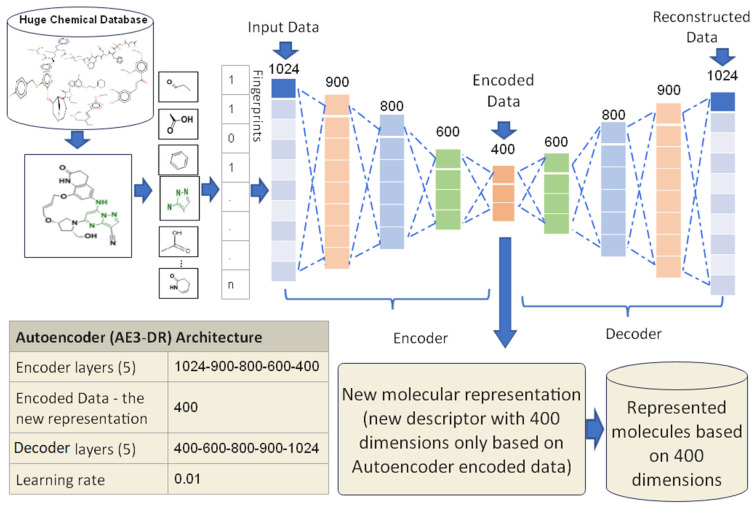
The AE3-DR proposed design.

The AE3-DR has been trained using five encoder layers in which the input data to the first layer of the encoder is the molecule vector with 1024 features. The remaining four hidden layers have 900, 800, 600, and 400 nodes, respectively. The size of the encode layer in this proposed case is 400, where it will be used as a new representation for the molecules, and the size of the decoder layers in this proposed case are 400, 600, 800, 900, and 1024 respectively. The last decoder layer is the reconstructed input data which is mostly like the input data. All molecules in the proposed datasets have been trained using the AE3-DR until they achieved a lesser error rate of the training than the proposed learning rate value, which is 0.01. The proposed design of AE3-DR is shown in Figure 6. The finished training output of AE3-DR is a new molecules representation with 400 dimensions which is only used for similarity search between molecules. The experimental results of AE3-DR have been presented in Section 5.

#### 3.3.4. Similarity Searching Based Autoencoder Molecular Representation Using Tanimoto Similarity Measure

Several similarity methods have been developed in virtual screening to calculate the similarity between the query and the molecular database. In this study, the continuous Tanimoto measure is utilized to calculate the similarity of the molecules in the represented datasets, which are based on three Autoencoder proposed cases. The continuous Tanimoto measure formula is expressed in Equation (7) where $S_{AB}$ is the similarity between molecules *A* and *B* and the molecules *A* and *B* are represented Autoencoder with new vectors ƒ of length N and N has a different length based on the size of the autoencoder code layer for all the proposed cases, where $f_{iA}$ is the value of the i fragments of molecule A and $f_{iB}$ is the value of the i fragments of molecule B.

(4) $$S_{AB}=\frac{\sum_{i=1}^{N}f_{iA}\;f_{iB}}{\sum_{i=1}^{N}{\left(f_{iA}\right)}^{2}+\sum_{i=1}^{N}{\left(f_{iB}\right)}^{2}+\sum_{i=1}^{N}f_{iA}\;f_{iB}}$$

## 4. Experimental Design 

The Autoencoder (AE) can be used as supervised and unsupervised training models. For the proposed model, we used the unsupervised Autoencoder for training all the molecules in the dataset in order to remove irrelevant and redundant features and generate a new representation of the molecules. The low dimensionality of the new molecular representation helps to improve the molecular similarity searching. The proposed models in the experiments are built using Keras.

**Figure 7:**
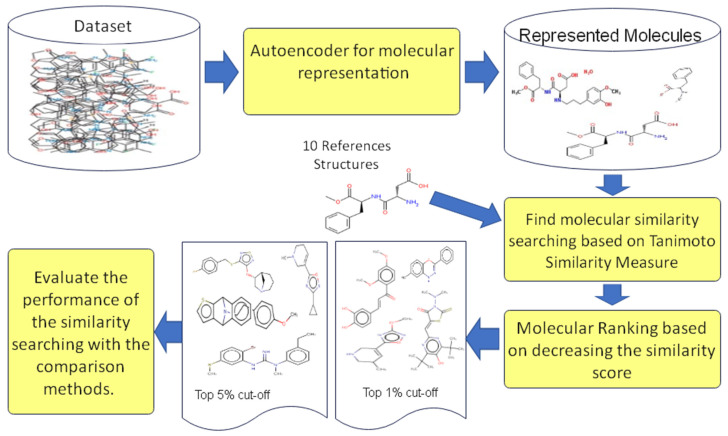
The Experimental design processes.

This study uses three different benchmark datasets that feature 2D structure representations to conduct simulated virtual screen searches to examine the effectiveness of the proposed molecular representations based on Autoencoder deep learning. The datasets are the MDDR (MDL Drug Data Report) from MDDR datasets. DS1, DS2, and DS3 datasets are used with different 2D fingerprints that consist of different bit strings length. The extended connectivity fingerprints count (ECFC), which consists of 1024 bits, is used to be an input to each proposed case of the Autoencoder. Moreover, each proposed case of Autoencoder was trained until it achieved an error rate of training lesser than the proposed learning rate and for all the molecules in the proposed datasets. For each proposed case, the output serves as a new low-dimensional molecular representation based on the encoded data layer. The new representations are used as a new descriptor for molecular similarity searching. Figure 7 depicts the experiment design steps used in the proposed study. 

The experiments were carried out using several similarity measures to provide a comparison result between all three proposed cases of Autoencoder with commonly used and conventional similarity measures such as the Tanimoto Similarity Method (TAN) [63], which is deemed as the baseline and standard similarity measure; Adapted Similarity Measure of Text Processing (ASMTP) [39]; and Quantum-Based Similarity Method (SQB) [38]. 

The simulated virtual screening studies were conducted by searching using ten reference structures selected at random from each activity class. All the previously mentioned similarity measures are applied with the selected references that were unified. The final output, derived from the similarity findings of all molecules in the database, is then ranked in decreasing order. This is usually done in ligand-based virtual screening to investigate and identify where the active compounds will appear in the ranked list. The presence of active compounds at the top of the ranking list demonstrates the effectiveness of the virtual screening techniques. A similar procedure is followed for all experiments performed on all datasets. The average retrieved output of the ten references’ query results mean is calculated in the 1% and 5% recall data cut-offs. Then, the average of the recall results of all classes is calculated to compare and evaluate the proposed measure with the standard measures. This process is repeated for all datasets and all experiments in this research.

### 4.1. Datasets

Evaluating the similarity searching methods requires chemical datasets that can carry out retrospective searches based on compounds of known activity. Therefore, several licensed datasets can be used to assign biological activities to chemical compounds applied on various algorithms for evaluation purposes. The MDL Drug Data Report (MDDR) dataset is one of the most widely used chemo-informatics databases for measuring the success of retrieving active chemical structures from similarity screens (Accelrys Inc.: San Diego, CA, USA, http://www.accelrys.com, accessed on 15 January 2020) [64] and has been used in several studies to validate ligand-based virtual screening methods [34,38,39,44]. The MDDR database consists of over 102,000 chemical compounds with hundreds of different activities, some of which are related to therapeutic areas such as antihypertensive, while others include specific enzymes such as Renin inhibitors. In contrast to other datasets that use unstructured text to represent activity, the MDDR dataset has a limited set of set activities. Table 1, Table 2 and Table 3 show the specifics of the dataset’s active compounds.

In this paper, the experiments were carried out using the MDDR dataset to simulate virtual screening to evaluate various methods in this work. All MDDR datasets utilized in this work are 2D structural representations converted to multiple fingerprint descriptors using Pipeline Pilot’s software [65]. The descriptors used in these research experiments are ECFC_4 (Extended Connectivity Counts). The MDDR dataset contains 102,516 active and inactive molecule data from the MDDR DS1, MDDR DS2, and MDDR DS3 datasets. The MDDR-DS1 comprises eleven distinct activity classes, some of which include structurally homogeneous actives (i.e., structurally diverse). On the other hand, the MDDR-DS2 dataset comprises ten homogeneous classes of activity, whereas the MDDR-DS3 dataset comprises ten heterogeneous classes of activity. The following Table 1, Table 2 and Table 3 provided each description for the datasets used.

The diversity level in each of the chosen sets of bioactivities can be estimated, using Pipeline Pilot software, through the matching of each chemical structure with every other structure in its activity class. The class diversity is calculated using ECFC 4 fingerprints and the Tanimoto coefficient. These findings were provided in the tables where the scores demonstrate that activity class “Vitamin D analogs”, as shown in Table 2, is the most homogeneous, while activity class “NMDA receptor antagonists”, as shown in Table 3, is the most diverse.

### 4.2. Evaluation Measures of the Performance

The performance of the similarity methods is measured using different evaluation methods. First, the screening is performed using the proportion of active compounds discovered within the top 1% and 5% of the ranking test set. Most virtual screening techniques commonly employ the top 1% and 5% to measure the recall of virtual screening methods [66].

The second measure involves the comparison of proposed methods against the benchmark approach. Over the past years, the searching benchmark approach in ligand-based virtual screening has been the Tanimoto similarity method. Additionally, several existing methodologies are available for the performance evaluation of the proposed methods. The methods are listed as the following:

- Tanimoto Similarity Method (TAN) [63]: is used to calculate both binary and distance similarity coefficients.
- Adapted Similarity Measure of Text Processing (ASMTP) [39] is a similarity measure based on ligand-based virtual screening. It has been generated to utilize the process of chemical structure databases for a textual database.
- Quantum-Based Similarity Method (SQB) [38] is a method for determining molecular similarity based on quantum mechanics. To improve the model’s performance, the approach concentrates on the complex pure Hilbert space of molecules.

The final important measure that can be used to evaluate the proposed methods is the Significance Test. The Kendall W test of concordance is one of the important measures that use the Significance Test for a quantitative method for measuring the performance of the similarity approach [67]. In particular, Kendall’s W was used to translate the coefficient of concordance, commonly referred to as the measure of agreement among raters. The assumption is based on, there is either a judge or a rater for each possible instance and that each variable is either an object or a person being judged. The overall rankings for each variable are determined accordingly. The range of Kendall’s W is between 0 (no agreement) and 1 (complete agreement). Assume that object *i* (search similarity method) is provided a rank $r_{i,j}$ by judge number *j* (activity class), where there are in total *n* objects and *m* judges. Thus, the total rank provided to the object *i* is:

(5) $$R_{i}=\overset{m}{\underset{j=1}{\sum }}r_{i,j}$$

and the mean value of these total rankings is

(6) $$\bar{R}\;=\frac{1}{n}\;\overset{n}{\underset{i=1}{\sum }}R_{i}$$

The sum of squared deviations, S, is defined as

(7) $$S=\overset{n}{\underset{i=1}{\sum }}{\left(R_{i}-\bar{R}\right)}^{2}$$

and then Kendall’s W is defined as

(8) $$W=\frac{12\;S}{m^{2}\left(n^{3}-n\right)}$$

The Kendall W test determines if a group of judges can reach similar conclusions regarding the rank of each set of objects and vice versa. An experiment is carried out as part of this study. Each of the dataset’s activity classes served as judges, while the recall rates of the various search models served as objects. The findings supplied the Kendall coefficient value and corresponding relevance levels, demonstrating whether the occurrences of the coefficient are due to chance. It was possible to provide an overall object ranking provided the value was important (cut-off values of both 1% and 5% were used).

## 5. Experimental Results and Discussion

The study results presented a new molecules representation based on autoencoder dimensionality reduction (AE_DR) to exploit the powerful ability to learn a feature representation of molecules from low-level encodings of a large corpus of chemical structures. In addition, the similarity score between the reference structures of the molecules and the entire represented molecules was calculated. As noted in Section 3.3, three different autoencoder architectures were proposed in this study (AE1-DR, AE2-DR, AE3-DR). The retrieval outcomes of all three proposed autoencoder cases were compared to different comparison approaches, including the Tanimoto Similarity Method (TAN), the Adapted Similarity Measure of Text Processing (ASMTP), and the Quantum-Based Similarity Method (SQB). For many years, the TAN coefficient has been employed in ligand-based virtual screening and has been deemed as a reference standard, whereas the others have only recently been applied with a similar aim of improving the performance of similarity searching. The overall experimental findings of the MDDR-DS1, MDDR-DS2, and MDDR-DS3 based on the new molecular representation are reported in Table 4, Table 5, Table 6, Table 7, Table 8 and Table 9, with cut-off values of 1% and 5%. The dataset’s activity class is represented in the first column of each table. The second, third, and fourth columns reflect the average recall achieved at the top 1% and top 5% ranking results for each activity class, using TAN, ASMTP, and SQB similarity metrics, respectively. The results achieved by the three proposed cases of the autoencoder approach are represented in the following columns. The average recall for the top 1% and top 5% of the rankings is presented at the end of each column, and the contrasted shaded cell counts for all techniques will be evaluated.

The recall values of MDDR-DS1 are shown in Table 4 and Table 5 for the 1% and 5% cut-off, respectively. Table 6 and Table 7 show the recall values of MDDR-DS2 results of the top 1% and 5%, respectively, and Table 8 and Table 9 report the retrieval recall results for MDDR-DS3 data sets of the top 1% and 5%, respectively. The last row of each table demonstrated the number of classes that achieved the best recall from each similarity measure. The best recall average values for each class are shaded. 

Table 4 and Table 5 summarizes the top 1% and top 5% recall rates for DS1, respectively. Based on findings, the three proposed cases of AE1-DR, AE2-DR, and AE3-DR surpassed both TAN and ASMTP at the 1% and 5% cut-off values. Meanwhile, the AE3-DR surpassed TAN, ASMTP, and SQB for the 1% and 5% cut-off. In the first case, the AE1-DR surpasses all TAN and ASMTP benchmark comparison approaches, with increases of 2% in mean recall values when compared to TAN and 1.78 in mean recall values when compared to ASMTP, respectively, for the top 1%. The findings for the top 5% are provided in Table 5, where it can be observed that the AE1-DR surpassed TAN by 2.54% mean recall values and ASMTP by 1.20% mean recall values. In the second case, the AE2-DR with DS1 surpassed the TAN and ASMTP benchmark similarity approaches, increasing mean recall values by 1.68% to TAN and 1.46% compared to ASMTP for the top 1%. The AE2-DR surpassed TAN and ASMTP in the top 5%, achieving 2.92% mean recall values and 1.58% mean recall values, respectively. Finally, compared to the TAN, ASMTP, and SQB benchmark methods, the third recommended case of AE3-DR yielded the best results. On the DS1 dataset, AE3-DR surpassed the other similarity techniques in the top 1%, with mean recall values increased by 2.64% to TAN, 2.42% when compared to ASMTP, and 0.49% when compared to SQB. The findings of the top 5% demonstrated that AE3-DR performed well, with mean recall values increasing by 3.96% compared to TAN, 2.62% when compared to ASMTP, and 0.96% when compared to SQB.

The DS2 dataset comprises ten homogeneous activity classifications. This dataset’s molecules are more similar to one another with lower diversity. In Table 6 and Table 7, the recall values for the three proposed cases were compared to those obtained using the TAN, ASMTP, and SQB benchmark techniques. The AE1-DR beats the TAN, and ASMTP benchmark similarity approaches for the top 1%, with increases of 16.85% mean recall values versus TAN and 4.47% mean recall values versus ASMTP. The AE2-DR for the top 1% with DS2 outperforms the TAN and ASMTP benchmark similarity methods, with increases of 17.95% mean recall values compared to TAN, and 5.57% mean recall values compared to ASMTP. Among all three proposed cases, the AE3-DR for the top 1% with DS2 demonstrated the best results compared to TAN, ASMTP, and SQB benchmark methods. The AE3-DR outperformed other similarity methods, with increases of 18.70% for mean recall value compared to TAN, 6.32% compared to ASMTP, and 0.57% compared to SQB. The results for the top 5% of the three proposed cases of AE1-DR, AE2-DR, and AE3-DR are provided in Table 7, where each case outperforms the TAN with increases of 15.86%, 16.79%, and 17.45%, respectively. The ASMTP and SQB, on the other hand, outperformed the proposed cases. The DS2 Dataset is more homogenous, with less important and redundant characteristics that might affect similarity performance. Therefore, compared to ASMTP and SQB, the performance of similarity searches using the new molecules’ representations based on low dimensions was worse. Thus, our work focuses on how to improve the similarity between molecules that are diverse and have low similarity values.

The DS3 dataset comprises ten heterogeneous activity classes with highly diverse molecules. Based on the findings, the DS3 dataset produces the best outcomes. Table 8 shows the top 1% retrieval results of AE1-DR, AE2-DR, and AE3-DR when compared to TAN and SQB benchmark methods, while Table 9 presents the retrieval results of the top 5% of AE1-DR, AE2-DR, and AE3-DR. The results of AE1-DR in the top 1% demonstrated increased performance, with improvements of 2.26% in mean recall values compared to TAN and 1.51% in mean recall values compared to SQB. While the AE1-DR performed well in the top 5% of results, it gained 2.86% of mean recall values compared to TAN and 3.29% of mean recall values compared to SQB. In the second case, the outcomes of AE2-DR with the top 1% performed better than the TAN, with improvements of 2.56% of mean recall values and 1.81% of mean recall values compared to SQB. While the AE2-DR fared better in the top 5% of results, with improvements of 3.18% mean recall values compared to TAN, and 3.61% mean recall values compared to SQB. Compared to benchmark methodologies, the AE3-DR findings outperformed the other two proposed cases. AE3-DR outperformed the benchmark similarity approaches for the top 1%, gaining 3.08% of the mean recall values compared to TAN and 2.33% of the mean recall values compared to SQB. While the AE3-DR fared better for the top 5% of results, with improvements of 3.63% of the mean recall values when compared to TAN and 4.06% when compared to SQB. 

Another important measure used to evaluate the performances of the proposed method is a quantitative approach known as the Significance Test. The Kendall W test of concordance, as previously discussed in Section 4.2, was employed as the Significance Test in this study. The Kendall W significance test determines if a group of raters makes comparable judgments about the ranking of a group of objects; the raters, in this case, are the activity classes, and the item to be ranked is represented by the recall rates of the various search methods. Kendall’s coefficient of concordance test aims to measure whether the output result of the virtual screening was random or if the proposed approach produced good results that enhanced the virtual screening efficiency. In this study, if the value was significant, for which the cut-off values of 1% and 5% were chosen, it is possible to rank the items overall. Table 10 summarizes the top 1% and top 5% ranks for the different similarity findings of the TAN, ASMTP, SQB, AE1-DR, AE2-DR, and AE3-DR based on the Kendall analysis for DS1, DS2, and DS3 with ECFC 4 fingerprints. Table 10 has columns labeled with the dataset type, recall%, Kendall W coefficient value, related probability, and ranking of each of the six approaches.

Kendall W tests have a range of 0 to 1, with 0 indicating no agreement and 1 indicating total agreement, and the associated probability (*p*) should be less than 0.05. Kendall W test values for DS1 were *p* = 0.00012 and *w* = 0.19 for the top 1% retrieval results, *p* = 0.03 and *w* = 0.11 for the top 5% retrieval results, *p* = 0.002 and *w* = 0.49 for the top 1% retrieval results, *p* = 0.001 and *w* = 0.61 for the top 5% retrieval results, and *p* = 0.000 for the DS3 dataset. Thus, the Kendall W test findings for the top 1% and top 5% of all datasets DS1, DS2 and DS3 indicate that the associated probability (*p*) is less than 0.05. This indicates that the AE1-DR, AE2-DR, and AE3-DR are statistically significant at both the 1% and 5% cut-offs. Thus, the overall ranking of techniques for DS1 with the top 1% is AE3-DR > SQB > AE1-DR > AE2-DR > ASMTP > TAN while for the top 5% is AE3-DR > SQB > ASMTP > AE2-DR > AE1-DR >TAN. As for the DS2, the overall ranking of techniques for the top 1% is AE3-DR > AE2-DR > SQB > AE1-DR > ASMTP > TAN while for the top 5% is SQB > ASMTP > AE3-DR > AE2-DR > AE1-DR >TAN. Next, for DS3, the overall ranking of techniques for the top 1% is AE3-DR > AE2-DR > AE1-DR > SQB > TAN while for the top 5% is AE3-DR > AE2-DR > AE1-DR > TAN > SQB.

As mentioned in the related works section, the Bayesian inference network (BIN) is the most popular method of machine learning used for molecular similarity searching that provided an interesting performance to the existing tools of similarity-based virtual screening. The BIN is particularly effective when the active molecules being sought have a high degree of structural homogeneity but has been found to perform less well with the structurally heterogeneous sets of molecules. The mean recall value results of BIN based on the MDDR-DS3 heterogeneous dataset is 10.55 with a cutting-off of 1 %, while the mean recall value results with cutting 5% is 17.81 [36]. In this paper, we aimed to improve the performance of similarity searching for the heterogeneous dataset, where the molecules are highly diverse and where the benchmarks methods failed to improve the performance of the LBVS similarity searching. Thus, we introduced an alternative approach for molecular representation called Autoencoder, which aims to remove irrelevant and redundant features that impact the performance of the similarity searching methods and produces a new molecules’ representation (new descriptor with low dimensions) used for improving the performance of the similarity searching. This helped overcome the limitations of the previous methods, such as BIN. For instance, the results of the Autoencoder cases AE1-DR, AE2-DR, and AE3-DR with the top 1% showed an improved performance using the mean recall values compared to BIN (12.01, 12.31, 12.83, and 10.55, respectively. Similarly, for the top 5%, AE1-DR, AE2-DR, and AE3-DR obtained better performance than BIN (21.29, 21.61, 22.06, and 17.81).

## 6. Conclusions

The selection of favorable reduced metric space without prior information is not easy. However, the use of new algorithms that can learn complex functions has opened up a new way of providing a lower-dimensional representation of the data without significant information loss. Autoencoders have proven extremely effective at reducing data dimensionality while preserving significant underlying features. Three proposed cases with different architectures of Autoencoder have been employed to remove irrelevant and redundant features and produce a new molecular representation with low dimensions. This new representation based on the low dimension is regarded as a new descriptor and used to enhance the recall of the similarity searching measures. The proposed cases resulted in a low dimensional molecular representation that preserved the significant underlying features. The represented datasets were utilized to calculate the molecular similarity search using the Tanimoto similarity measure. The experiments performed in this study using the MDDR benchmark dataset demonstrated that virtual screening of chemical databases using ligands is more cost-effective than other techniques. The screening and evaluation findings indicated that the proposed method outperforms other similarity search methods, including Tanimoto coefficient (TAN), Adapted Similarity Measure for Text Processing (AS-MTP), and Quantum-Based Similarity (QBS) approaches. The screening investigation demonstrated that the new approaches outperformed the existing methods. The performance of the three proposed cases featuring structurally heterogeneous datasets (MDDR-DS1, MDDR-DS3), in particular, outperformed current approaches used in prior work to improve molecular similarity searches.

## Figures and Tables

**Table 1:** The MDDR-DS1 structure activity classes.

Activity Class	Active Molecules	Activity Index	Pairwise Similarity
Renin inhibitors	1130	31,420	0.290
HIV protease inhibitors	750	71,523	0.198
Thrombin inhibitors	803	37,110	0.180
Angiotensin II AT1 antagonists	943	31,432	0.229
Substance P antagonists	1246	42,731	0.149
5HT3 antagonist	752	06233	0.140
5HT reuptake inhibitors	359	06245	0.122
D2 antagonists	395	07701	0.138
5HT1A agonists	827	06235	0.133
Protein kinase C inhibitors	453	78,374	0.120
Cyclooxygenase inhibitors	636	78,331	0.108

**Table 2:** The MDDR-DS2 structure activity classes.

Activity Class	Active Molecules	Activity Index	Pairwise Similarity
Adenosine (A1) agonists	207	07707	0.229
Adenosine (A2) agonists	156	07708	0.305
Renin inhibitors	1130	31,420	0.290
CCK agonists	111	42,710	0.361
Monocyclic β-lactams	1346	64,100	0.336
Cephalosporins	113	64,200	0.322
Carbacephems	1051	64,220	0.269
Carbapenems	126	64,500	0.260
Tribactams	388	64,350	0.305

**Table 3:** The MDDR-DS3 structure activity classes.

Activity Class	Active Molecules	Activity Index	Pairwise Similarity
Muscarinic (M1) agonists	900	09249	0.111
NMDA receptor antagonists	1400	12,455	0.098
Nitric oxide synthase inhibitors	505	12,464	0.102
Dopamine β-hydroxylase inhibitors	106	31,281	0.125
Aldose reductase inhibitors	957	43,210	0.119
Reverse transcriptase inhibitors	700	71,522	0.103
Aromatase inhibitors	636	75,721	0.110
Cyclooxygenase inhibitors	636	78,331	0.108
Phospholipase A2 inhibitors	617	78,348	0.123
Lipoxygenase inhibitors	2111	78,351	0.113

**Table 4:** The Retrieval results of the top 1% for the MDDR-DS1 dataset.

Activity Index	TAN	ASMTP	SQB	AE1_DR	AE2_DR	AE3_DR
31,420	69.69	73.84	73.73	71.31	70.43	70.99
71,523	25.94	15.03	26.84	28.37	25.37	25.85
37,110	9.63	20.82	24.73	21.40	21.90	20.92
31,432	35.82	37.14	36.66	41.34	40.71	41.04
42,731	17.77	19.53	21.17	19.23	17.67	22.03
06233	13.87	10.35	12.49	13.01	14.04	14.87
06245	6.51	5.50	6.03	6.03	7.78	7.08
07701	8.63	7.99	11.35	9.87	8.91	12.31
06235	9.71	9.94	10.15	10.71	11.07	10.49
78,374	13.69	13.90	13.08	11.91	12.04	13.74
78,331	7.17	6.89	5.92	7.23	7.07	8.14
**Mean**	19.86	20.08	22.01	21.86	21.54	**22.50**
**Shaded** **cells**	0	2	1	2	2	**4**

**Table 5:** The Retrieval results of top 5% for MDDR-DS1 dataset.

Activity Index	TAN	ASMTP	SQB	AE1_DR	AE2_DR	AE3_DR
31,420	83.49	86	87.75	85.8	85.03	87.08
71,523	48.92	51.33	60.16	55.21	57.22	56.41
37,110	21.01	23.87	39.81	43.53	42.17	41.79
31,432	74.29	76.63	82	78.72	80.40	80.12
42,731	29.68	32.9	28.77	27.04	26.03	27.04
06233	27.68	26.2	20.96	23.8	24.11	25.19
06245	16.54	15.5	15.39	19.76	21.17	21.07
07701	24.09	23.9	26.90	25.21	24.78	26.25
06235	20.06	23.6	22.47	22.08	21.91	24.17
78,374	20.51	22.26	20.95	18.19	19.88	23.74
78,331	16.2	15	10.31	11.07	11.9	13.19
**Mean**	34.77	36.11	37.77	37.31	37.69	**38.73**
**Shaded** **cells**	2	1	**3**	0	1	**3**

**Table 6:** The Retrieval results of top 1% for MDDR-DS2 dataset.

Activity Index	TAN	ASMTP	SQB	AE1_DR	AE2_DR	AE3_DR
07707	61.84	67.86	72.09	70.15	73.18	73.46
07708	47.03	97.87	95.68	95.73	97.57	98.75
31,420	65.10	73.51	78.56	73.75	75.17	74.04
42,710	81.27	81.17	76.82	80.12	83.03	82.01
64,100	80.31	86.62	87.80	86.19	88.17	87.79
64,200	53.84	69.11	70.18	67.61	67.02	69.08
64,220	38.64	66.26	67.58	67.96	66.74	67.19
64,500	30.56	46.24	79.20	74.04	76.02	79.72
64,350	80.18	68.01	81.68	81.96	81.77	83.09
75,755	87.56	93.48	98.02	97.26	97.08	98.15
**Mean**	62.63	75.01	80.76	79.48	80.58	**81.33**
**Shaded** **cells**	0	0	2	1	2	**5**

**Table 7:** The Retrieval results of top 5% for MDDR-DS2 dataset.

Activity Index	TAN	ASMTP	SQB	AE1_DR	AE2_DR	AE3_DR
07707	70.39	76.17	74.22	73.33	77.78	80.24
07708	56.58	99.99	100	97.9	98.03	99.28
31,420	88.19	95.75	95.24	92.08	94.11	95.22
42,710	88.09	96.73	93	91.06	91.27	92.71
64,100	93.75	98.27	98.94	98.90	97.41	97.85
64,200	77.68	96.16	98.93	93.80	94.80	95.90
64,220	52.19	94.13	90.9	91.5	92.09	92.33
64,500	44.8	90.6	92.72	89.04	91.08	91.07
64,350	91.71	98.6	93.75	91.11	92.44	90.9
75,755	94.82	97.27	98.75	98.08	97.09	97.19
**Mean**	75.82	**94.36**	93.61	91.68	92.61	93.27
**Shaded** **cells**	0	4	**5**	0	0	1

**Table 8:** The Retrieval results of top 1% for MDDR-DS3 dataset.

Activity Index	TAN	SQB	AE1_DR	AE2_DR	AE3_DR
09249	12.12	10.99	15.01	16.03	17.76
12,455	6.57	7.03	7.88	9.17	6.77
12,464	8.17	6.92	11.12	12.50	12.04
31,281	16.95	18.67	17.66	17.75	16.5
43,210	6.27	6.83	9.76	9.07	10.90
71,522	3.75	6.57	7.19	9.14	9.02
75,721	17.32	20.38	22.29	21.66	23.90
78,331	6.31	6.16	6.09	5.06	8.98
78,348	10.15	8.99	9.11	6.89	6.40
78,351	9.84	12.5	14.02	15.78	16.06
**Mean**	9.75	10.50	12.01	12.31	**12.83**
**Shaded** **cells**	1	1	0	3	**5**

**Table 9:** The Retrieval results of top 5% for MDDR-DS3 dataset.

Activity Index	TAN	SQB	AE1_DR	AE2_DR	AE3_DR
09249	24.17	17.8	26.08	26.02	25.79
12,455	10.29	11.42	14.85	15.86	14.99
12,464	15.22	16.79	19.76	20.74	19.78
31,281	29.62	29.05	32.33	33.19	35.01
43,210	16.07	14.12	19.11	20.22	19.55
71,522	12.37	13.82	15.44	15.07	16.06
75,721	25.21	30.61	33.71	34.45	35.33
78,331	15.01	11.97	13.22	13.10	14.12
78,348	24.67	21.14	20.87	20.98	21.89
78,351	11.71	13.30	17.50	16.45	18.08
**Mean**	18.43	18.00	21.29	21.61	**22.06**
**Shaded** **cells**	2	0	1	3	**4**

**Table 10:** The Rankings of TAN, ASMTP, SQB, AE1-DR, AE2-DR, and AE3-DR approaches Based on Kendall W Test Results: DS1, DS2, and DS3 at top 1% and top 5%.

Data Set	Recall Cut-Off	W	P	Mean Rank					
				TAN	ASMTP	SQB	AE1_DR	AE2_DR	AE3_DR
DS1	1%	0.19	0.00012	1.56	1.64	2.59	2.95	2.55	**3.737**
DS1	5%	0.11	0.03	1.73	2.55	2.82	2.05	2.36	**3.5**
DS2	1%	0.49	0.002	0.4	1.7	3.2	2.4	3.2	**4.1**
DS2	5%	0.61	0.001	0.2	**4.4**	**4.4**	1.8	2.6	3.1
DS3	1%	0.23	0.0001	1	Not used	1.4	2.3	2.6	**2.7**
DS3	5%	0.47	0.0011	1.1	Not used	0.7	2.2	2.7	**3.3**

## Data Availability

The MDL Drug Data Report (MDDR) dataset is owned by www.accelrys.com, accessed on 15 January 2020. A license is required to access the data.
